# In Vitro Interaction between *Mycoplasma agalactiae* and Small Ruminants’ Endogenous Bacterial Strains of *Enterococcus* spp. and Coagulase-Negative *Staphylococcus*

**DOI:** 10.3390/microorganisms12020406

**Published:** 2024-02-17

**Authors:** Marion Toquet, Esther Bataller, Raquel Toledo-Perona, Jesús Gomis, Antonio Contreras, Antonio Sánchez, Estrella Jiménez-Trigos, Ángel Gómez-Martín

**Affiliations:** 1 Microbiological Agents Associated with Animal Reproduction (ProVaginBIO) Research Group, Departamento Producción y Sanidad Animal, Salud Pública Veterinaria y Ciencia y Tecnología de los Alimentos, Facultad de Veterinaria, Universidad Cardenal Herrera-CEU, CEU Universities, Carrer Tirant lo Blanc, 7, 46115 Valencia, Spain; marion.toquet1@uchceu.es (M.T.); esther.bataller@uchceu.es (E.B.); raquel.toledoperona@uchceu.es (R.T.-P.); jesus.gomis1@uchceu.es (J.G.); estrella.jimenez@uchceu.es (E.J.-T.); 2 Departamento de Sanidad Animal, Facultad de Veterinaria, Universidad de Murcia, Campus de Espinardo, 30100 Murcia, Spain; acontrer@um.es (A.C.); asanlope@um.es (A.S.)

**Keywords:** *Mycoplasma agalactiae*, *Staphylococcus*, *Enterococcus*, milk, vagina, microbial interaction, sheep, goat

## Abstract

Recently, an antimicrobial effect on *Mycoplasma agalactiae* (Ma), the main etiological agent of contagious agalactia (CA), was reported in vitro with strains of *Enterococcus* spp. from ovine and caprine milk. The aim of this work was to evaluate the interaction of Ma with the same *Enterococcus* spp. isolated from other anatomical locations (vagina) and other bacterial populations present in milk, such as coagulase-negative staphylococci (CNS). The vaginal *Enterococcus* strains and the raw milk CNS were isolated from sheep and goats. Experimental in vitro conditions were prepared to assess the growth of Ma with and without the presence of these strains. The selected vaginal strains were identified as *Enterococcus (E.) hirae* and *E. mundtii,* and the strains of CNS were identified as *Staphylococcus petrasii*. Different interactions of Ma with ovine and caprine wild vaginal strains of *Enterococcus* and dairy strains of CNS are described for the first time: Ma can grow exponentially during 15 h with the selected strains, although with certain strains, its optimal growth can be negatively affected (*p* < 0.05). The colonization and/or excretion of Ma could, therefore, be influenced by certain endogenous bacterial strains. Our results increase the knowledge about possible bacterial ecology dynamics surrounding CA.

## 1. Introduction

Contagious agalactia (CA) is a mycoplasmosis produced by up to four species of mycoplasmas: *Mycoplasma (M) agalactiae* (Ma), *M. mycoides* subsp. *Capri* (Mmc), *M. capricolum* subsp. *capricolum* and *M. putrefaciens* (CA-mycoplasmas). *Mycoplasma agalactiae* is the most relevant species in the caprine species and the only one involved in CA in the ovine species. On the one hand, the repercussions of this disease are important, especially in dairy herds in endemic areas due to mammary tropism. Indeed, it is common to see a reduction in milk production in the affected animals [1,2] and the economic devaluation of milk in clinical outbreaks due to the high somatic cell counts [3]. On the other hand, the ability of CA-mycoplasmas, such as Ma, Mmc and *M. putrefaciens*, to colonize and/or damage the reproductive tract has been reported, leading to lesions such as vulvovaginitis, salpingitis and cystic catarrhal metritis in females or testicular degenerations and balanoposthitis in males. The vagina, urethra, testicles and bulbourethral glands are locations where some of these species have been identified [4,5,6,7,8,9]. The ability of Ma and Mmc to be excreted naturally in the ejaculate has also been described [9,10] to survive in diluted semen and even, in the case of Mmc, to affect their sperm viability [11], which could imply undervalued reproductive repercussions [5,9]. Based on all this, the mammary gland and the reproductive tract are places for which CA-mycoplasmas have a tropism and in which it seems interesting to improve the knowledge of those factors that favor or hinder this colonization in order to improve fight strategies against CA.

The use of vaccination and antibiotic therapy is common to control and prevent this disease [2]. Nevertheless, neither of the strategies eliminates the pathogen, and herds can become chronically infected with the presence of long-term shedders [12]. However, other alternative antimicrobial strategies have been suggested. The sensitivity of Ma and Mmc to pH below those usually used in cultures was evidenced in diluted goat semen and suggested as a possible antimicrobial strategy [11]. In this sense, lactic acid bacteria (LAB) are a bacterial community, naturally present in the reproductive tract and mammary gland of ruminants, capable of acidifying the pH [13]. These have been reported as in vitro antimicrobial strategies against *M. bovis* with an antimicrobial effect statistically similar to that of some antibiotics due to their ability to reduce the pH of their environment [14,15]. Recently, the first antibacterial effects of caprine and ovine LAB strains against Ma were described. Specifically, two species of *Enterococcus*, *E. hirae* and *E. mundtii*, previously isolated from raw sheep and goat milk, respectively, could represent a possible antimicrobial strategy not previously contemplated for the control of CA [16]. However, the question remains as to whether this antimicrobial effect against Ma is exclusive to *Enterococcus* strains from the milk of small ruminants or whether this antagonism could occur with LAB strains in other anatomical locations such as the vagina, where Ma [6,7] and LAB can be present [17,18,19,20], and therefore, there is a possibility that they coexist and interact with each other in this anatomical location.

*Enterococcus* spp. is a LAB of enteric origin [21], and so far, it has been the only bacterial genus for which an antibacterial effect against Ma has been tested and reported [16]. This bacterial genus has been able to colonize an important variety of habitats, thanks to the plasticity of their genome, such as the milk of ruminants through their presence in milking stations [22,23]. They are very common in small ruminants’ milk [24,25], and although their presence in the ewe’s vagina has been detected, they have only been scarcely described [18,20]. To the authors’ knowledge, there are no data available regarding the presence of enterococci in the vagina of goats as no metagenomic studies have been published. Although they can simply be commensal microorganisms, *Enterococcus* spp. can also act as opportunistic pathogens, especially in nosocomial infections. Most clinical infections are due to *E. faecalis* and *E. faecium*, and their growing antibiotic resistance, especially to vancomycin, has become a major concern [26]. Nevertheless, the genus *Enterococcus* also presents a wide range of probiotic strains producing bacteriocins [27].

On the other hand, since Ma must interact with all kinds of bacterial communities present in the mammary gland, this raises the need to study how Ma behaves with other bacterial populations naturally present in the mammary gland. *Staphylococcus* spp. is one of the principal etiological agents of intramammary infections (IMI) in small ruminants [2,28,29], and coagulase-negative staphylococci (CNS) are considered the main cause of subacute mastitis [28,30]. Nevertheless, the presence and abundance of this genus have also been described in several metagenomic studies of the caprine milk microbiota in healthy goats and ewes [25,31,32,33,34,35]. The prevalence of staphylococcal mammary carriage (i.e., the presence of the microorganism but no inflammation) has been reported to be around 6.5% in ewes [36]. In a recent study, *Staphylococcus* spp. were isolated from clinically healthy goats (45.9%); 72.3% of the isolates were identified as CNS, and the remaining 27.7% were identified as *S. aureus* [37]. Another study suggested that the staphylococcal bacterial community can produce inflammation in the mammary gland, acting as opportunist pathogens in case of dysbiosis, although they could also be a defense barrier against the entrance of pathogens [38].

Based on the above, *Staphylococcus* spp. and LAB are bacterial populations with which Ma can interact in the mammary gland and vagina of small ruminants. Overall, no data are available regarding the inhibition of mastitis agent by other potential mastitis pathogens in small ruminants or their potential synergy. Furthermore, no studies are available about the antimicrobial potential of vaginal LAB strains against Ma. Therefore, there is a need to carry out new studies about the influences of the bacterial ecology surrounding CA. The first objective of this study was to try to isolate LAB from the vagina of healthy sheep and goats to later study the possible in vitro effect that they could have on the viability of Ma. The second objective of this study was to evaluate in vitro the viability of Ma and caprine wild strains of CNS obtained from raw milk of healthy goats when both concur in goat milk.

## 2. Materials and Methods

### 2.1. Ethics Approval and Consent to Participate

The Animal Experimentation Ethics Committee (CEEA) of the CEU Cardenal Herrera University reviewed the methods employed in this study to manipulate the animals to obtain biological samples. Following the Spanish Royal Decree 53/2013, the committee said they considered that the method is exempt from ethical approval by the authorized body since it does not include any activity considered a procedure on animals (CEEA report 20/006).

### 2.2. Bacterial Strains Isolation and Selection

For the isolation of wild LAB of ovine and caprine origin, vaginal swabs were taken from a total of 88 animals from 11 different ovine and caprine farms located in different provinces of the Autonomous Communities of Castilla-La Mancha, Valencia and Andalusia, located in the east and south of Spain. The animal flocks included four dairy goat herds, three dairy sheep herds, one meat goat herd and three meat sheep herds. All the characteristics of the sampled herds are available in Table 1. In each flock, vaginal swabs (Deltalab, Eurotubo^®^, Barcelona, Spain) were collected from eight animals in lactation, without any clinical signs, in which a California Mastitis Test (KerbaTEST, KERBL, Albert Kerbl GmbH, Burbach, Germany) was performed to rule out any animal with mastitis. The vulvar area was disinfected with 2% chlorhexidine prior to sample collection, and the samples were obtained by opening the vulva and carefully introducing the sterile swab into the vaginal tract without touching any other anatomical structure to avoid contamination. The swabs were then refrigerated and transported to the laboratory of the ProVaginBIO group (University CEU Cardenal Herrera) in less than 24 h.

Each swab was pre-cultured in Man, Rogosa and Sharpe (MRS) broth (Scharlau, Scharlab S.L., Barcelona, Spain) for 24 h at 37 °C, and then 10 μL of the pre-culture was inoculated on MRS agar (Scharlau, Scharlab S.L., Barcelona, Spain) [39]. The plates were incubated in anaerobiosis for 48 h at 37 °C. The isolates that grew on MRS agar were frozen in cryotubes with MRS broth and 50% glycerol and kept at −80 °C. The bacterial isolates were later tested in Columbia Agar with 5% sheep blood (BD^TM^, Becton, Dicksinson and Company, Madrid, Spain) to rule out strains producing hemolysis. Preliminarily selected strains were a posteriori tested for their growth in a specific medium for mycoplasmas culture (PH medium) [8] as previously described [16].

The two strains of CNS, 227A (OR289671) and 332B (OR289672), used in this study belonged to a collection of the ProVaginBIO investigation group of University CEU—Cardenal Herrera in Valencia, Spain—and their sequences are available in the GenBank repository (SUB13689910). They were previously isolated from raw milk of apparently healthy dairy (strain 332B) and meat (strain 227A) goats and identified using 16S rRNA sequencing and Basic Local Alignment Search Tools (BLAST, NCBI, Bethesda, USA) as *Staphylococcus* sp. strain Marseille P-8196 or *Staphylococcus petrasii* (query cover: 100%; percentage of identification: 99.88%) [16]. Coagulase test was performed using Rabbit Coagulase Plasma (BD BB^TM^) and came back negative for both strains. The strains were isolated from milk negative for California Mastitis Test. Strain 332B was isolated from herd I (Table 1), which was suffering a clinical outbreak of CA at the time of sampling, with the presence of symptoms such as mastitis in some animals. Strain 227A was isolated from herd E, which did not have any history of CA outbreaks.

### 2.3. Identification of Bacterial Strains

The vaginal LAB strains pre-selected for the in vitro experiments were identified by the amplification and sequencing of the marker gene 16S rRNA using Sanger technology. For each sample, the 16S rRNA gene was amplified using the primer pair 27F/1492R. Amplicons were sequenced using Sanger technology. Raw demultiplexed forward and reverse reads were processed using a modified version of the Automated Sanger Analysis Pipeline (ASAP) [40]. The analysis consisted of the following steps:

1. Biopython was used to convert input chromatogram sequences (.ab1) into FASTQ files [41];
2. Seqtk v1.3-r106 was used to trim sequences by Phred Quality Score under Q50;
3. Both forward and reverse quality trimmed reads were merged using the merger tool from European Molecular Biology Open Software (EMBOSS) v.EMBOSS:6.6.0.0 [42];
4. In order to assign the genomes to species, merged sequences were used as query for a BLASTn search [43] against the nt database from NCBI.

To obtain a more precise identification of strain CNS 227A, the complete genome was analyzed. DNA was extracted, and its purity was checked by sequencing the 16S rRNA gene (primers 27F/1492R) using Sanger technology. Genomic DNA libraries of 600 bp size were prepared, and sequencing was carried out using Illumina NextSeq paired ends (150 × 2 bp). Quality control of raw demultiplexed forward and reverse reads was performed using multiple tools. Briefly, descriptive stats of read quality were calculated using FastQC v0.11.8 and summarized with MultiQC v1.0 [44]. Then, adapters in 5′ ends were removed, and reads shorter than 75 nt were filtered using Trimmomatic v0.39 [45]. Trimmed paired reads were assembled using SPAdes v3.15.4 [46] using k-mers of 21, 33, 55, 77 bp long. Quality control of the assembly was performed using a custom script, and scaffolds shorter than 1000 bp long were removed from the analysis. Filtered scaffolds were annotated using Prokka v1.14.6 [47]. In order to assign a species classification to each genome, the CAMITAX pipeline was used [48].

### 2.4. In Vitro Experiments

We performed a total of four experiments: one for each isolated bacterial strain of both LAB (321A, 344A) and both staphylococci (227A, 332B). We followed the protocol described by [16], adapted from a methodology previously described [14,15]. Each of the four experiments was repeated in three independent replicates.

On the one hand, for the interaction study of Ma and LAB vaginal strains, three experimental conditions (C1, C2, C3, Table 2) plus one negative control (C4, Table 2) were prepared in PH medium.

On the other hand, for the study of the interaction of Ma with CNS, ten experimental conditions (C1-C10, Table 3) were prepared with semi-skimmed UHT goat milk (GM) or specific medium for the isolation of mycoplasma (PH) plus two negative controls (C11 and C12, Table 3) in Eppendorf-type tubes of 1.5 mL capacity. A dose of a commercial probiotic (L2) was also added as a positive control (C2, C3, C7 and C8) since its efficacy against Ma in GM has been demonstrated [16].

### 2.5. Inocula Preparation for the In Vitro Experiment

The Ma inoculum was prepared using the reference strain (PG2, NCTC10123) in PH medium with ampicillin [8] following a protocol previously described. An approximate concentration of 10^9^ CFU/mL was obtained [16].

The staphylococci and LAB inocula were prepared according to the methodology previously described [16]. Their concentrations were as follows: 3.7 × 10^7^ CFU/mL (227A), 1.0 × 10^9^ CFU/mL (332B), 2.7 × 10^8^ CFU/mL (321A) and 1.9 × 10^8^ CFU/mL (344A).

The L2 inoculum was prepared with an approximate concentration of 3.24 × 10^8^ CFU/mL, following a methodology previously described [14,15].

### 2.6. pH Determination

The pH of every condition was measured with a calibrated pH meter (SensION^TM^ + pH3, Hach, LPV2000.98.0002) after 15 min of incubation (T0) and after 15 h (T15). The electrode was disinfected with detergent, alcohol and sterile distilled water between the measurement of each condition to avoid contamination.

### 2.7. Bacterial Viability

To assess the bacterial viability, concentrations (CFU/mL) of Ma and LAB were determined at T0 and T15 following methodologies previously described [16,49]. The viability of *Staphylococcus* was assessed using the same protocol employed for the determination of LAB concentration.

### 2.8. Statistical Analysis

Counts of Ma, staphylococci and LAB were transformed as log (1 + C), where C was the count obtained (CFU/mL) for each analytical condition and organism. Statistical analysis was performed using a general linear procedure implemented in the program Statistical Analysis System Institute (SAS), using the following model: Y_ijk_ = μ + S_i_ + C_j_ + T_k_ + CT_jk_ + e_ijk_, where Y_ijk_ = pH and log CFU/mL of Ma and log CFU/mL of LAB or *Staphylococcus* in each strain studied (321A, 344A, 227A and 332B); μ = mean; S_i_ = sample effect; C_j_ = effect of analytical conditions; T_k_ = effect of time; CT_jk_ = effect of the interaction between the analytical condition and time; and e_ijk_ = residual effect.

## 3. Results

### 3.1. Selection and Identification of the Vaginal Strains

The number of isolated bacterial vaginal strains per herd and the selected ones for the in vitro experiment are shown in Table 4. Nine vaginal strains had good growth in a PH medium (>10^7^ CFU/mL) after 20 h of incubation. None of them produced hemolysis in Columbia Agar with 5% sheep blood. The best two strains (with the highest growth) were 321A and 344A. Strain 321A was isolated from herd H, and strain 344A was isolated from herd I, the same herd as CNS strain 332B, which suffered a clinical outbreak of CA at the time of sampling.

Strain 321A was identified as *E. hirae* (OR289673), and strain 344A was identified as *E. mundtii* (OR289674). Both strains are available in the GenBank repository (SUB13689910). The complete genome of CNS strain 227A was sequenced and assembled, with a taxonomic assignment result corresponding to *S. petrasii* (BioProject ID PRJNA1054492), and is available in the GenBank repository (SUB14096682).

### 3.2. Enterococcus spp. and M. agalactiae Interaction

In the proposed in vitro model, and for both *Enterococcus* strains, the condition itself, the time and the interaction between condition and time had a significant effect on the pH (*p* < 0.05). For *E. hirae* strain 321A, the condition itself, the time and the interaction between condition and time had a significant effect on the log CFU/mL of Ma (*p* < 0.001), while for *E. mundtii* strain 344A, only the factor time had a significant effect on the log CFU/mL of Ma *(p* < 0.001). The factor condition and the factor time contributed significantly to the observed log CFU/mL of *E. hirae* 321A (*p* < 0.05), while no factor significantly contributed to the observed log CFU/mL of *E. mundtii* 344A.

Table 5 presents the evolution of the pH and the viability of Ma per condition over time for the experiment with strain 321A. When on its own in the PH medium (C1), Ma concentration significantly increased (*p* < 0.001). When concurring with *E. hirae* 321A (C3), Ma concentration significantly increased (*p* < 0.001), although less than in C1 (*p* < 0.001). The average concentration of *E. hirae* 321A at T0 (C2 and C3) was 8.53 log CFU/mL, while it significantly decreased to 8.31 log CFU/mL at T15 (*p* < 0.05). The concentration of strain 321A was significantly superior on its own (C2), with an average of 8.55 log CFU/mL compared to 8.29 log CFU/mL when interacting with Ma (C3) (*p* <0.05). The pH of every condition at T15 was significantly lower than the pH of the control condition (C4).

The evolution of the pH and the viability of Ma per condition over time for the experiment with strain 344A are shown in Table 6. Ma average concentration (C1 and C3) significantly increased from 6.923 log CFU/mL at T0 to 8.402 at T15 (*p* < 0.001), and the presence of strain 344A had no influence. The pH was significantly lower at T15 in every condition compared to the pH of the control condition (C4). The concentration of strain 344A did not significantly vary.

### 3.3. Coagulase-Negative Staphylococci and M. agalactiae Interaction

In the proposed in vitro model, and for each CNS strain studied, the condition itself, the time and the interaction between condition and time had a significant effect (*p* < 0.001) on the pH and the log CFU/mL of Ma. No factor contributed significantly to the observed log CFU/mL of L2 (C2, C3, C7 and C8), while only the factor condition contributed significantly to the observed log CFU/mL of staphylococci for the strain 227A (*p* < 0.05).

Table 7 presents the evolution of the pH and the viability of Ma for the experiment with strain 227A. In favorable conditions (C1 and C6), Ma concentration significantly increased (*p* < 0.001) over 1 log CFU/mL at T15, and the pH showed stable values of over 6.60 in GM and over 7.33 in the PH medium. There was a significant increase in Ma concentrations with the presence of strain 227A at T15 in both GM (C5) and PH medium (C10), although these concentrations were significantly lower (*p* < 0.05) than the concentrations reached by Ma at T15 when *Staphylococcus* was not present (C1 and C6). The pH decreased significantly in every condition where *Staphylococcus* was present (C4, C5, C9 and C10) and in conditions with L2 in GM (C2 and C3). The average concentration of CNS strain 227A was significantly higher in conditions with GM (8.49 and 8.37 LOG CFU/mL for C4 and C5, respectively) than in conditions with PH medium (7.96 and 7.81 LOG CFU/mL for C9 and C10, respectively). L2 was able to completely inhibit Ma at T15 in GM (C3).

The evolution of the pH and the viability of Ma over time for the experiment with strain 332B are shown in Table 8. In favorable conditions (C1 and C6), Ma concentration significantly increased, and the pH showed stable values of over 6.22 in GM and over 7.35 in the PH medium. Conditions with both Ma and the strain 332B in GM (C5) and PH medium (C10) had a significant increase in the concentration of Ma at T15 with a significant decrease in the pH in GM (C5) and a stable pH in the PH medium (C10). L2 was able to significantly reduce the pH (C4 and C5) and completely inhibit Ma (C5) in GM.

## 4. Discussion

Since Ma has the ability to colonize the mammary gland and the vagina [9] and LAB and *Staphylococcus* spp. seem to be part of the microbiota of these anatomical sites [18,19,20,25,50], the present study describes the in vitro interactions between Ma and wild strains of CNS and *Enterococcus* spp. isolated from raw milk and vagina, respectively, of clinically healthy small ruminants. Our results suggest that when cohabiting in vitro with some strains of both bacterial genera, Ma can find a more hostile environment for its growth. Although this interaction did not inhibit the growth of the pathogen, it did have a negative influence on its optimal concentration at T15 and, therefore, poses a risk for Ma that could not reach infective concentrations in certain anatomical locations or secretions from an infected host. These results contribute to the knowledge regarding the bacterial ecology that surrounds CA. Evidence on the hitherto unknown influence on Ma viability exerted by other bacteria naturally present in healthy sheep and goats is offered, and this suggests the possibility of discovering new strategies and alternatives to antibiotics in the fight against this disease.

On the one hand, Ma has been detected in the vagina of small ruminants [6,7], where the near-neutral pH has been associated with a low abundance of LAB [17]. In our opinion, this offers a favorable environment for Ma and suggests that the vagina could represent a possible ecological niche of Ma in small ruminants. Indeed, the genus *Mycoplasma* has recently been described as the second most abundant in the vagina of ewes [20]. Although the presence of LAB in the vagina of small ruminants seems sparse, their use and the necessity to study them have been suggested in small ruminants due to their well-known antimicrobial potential [18,20,51]. Their antibacterial potential in vitro against *Mycoplasma* spp., using the same L2 inoculum employed in this study from a human commercial probiotic made of different *Lactobacillus* spp., has been reported for the first time against *M. bovis* [14,15] and later against Ma [16]. Nevertheless, the host-specific nature of LAB strains [52] would suggest the need to develop probiotics made of bacterial strains isolated from the same specific species for which the use is intended [25]. Our study reports the presence of two LAB species, *E. hirae* and *E. mundtii*, in the vagina of ovine and caprine animals, respectively, and therefore confirms that these two bacterial species are part of the vaginal microbiota of healthy small ruminants, as their presence was reported at a low prevalence in a previous study using metagenomics [18]. The two vaginal bacterial strains isolated in the present study came from herds located in an endemic area of CA in Spain, and in fact, strain 344A was isolated from a clinically healthy goat in a flock affected by an outbreak of CA. Given these circumstances, it is probable that interactions between Ma and LAB, such as the ones employed in this study, naturally occur in the vagina of small ruminants. This motivates the necessity to evaluate their in vitro effects on Ma.

Our study showed how the exponential growth of Ma in PH (C1, Table 5) was negatively influenced by the presence of *E. hirae* 321A (C3, Table 5). *Enterococcus* spp., while belonging to LAB, are not generally recognized as safe (GRAS) microorganisms, although some strains have been approved as probiotics or feed supplements for animals and humans [53]. In a previous study, three strains belonging to the genus *Enterococcus* (*E. mundtii* 33B, *E. hirae* 120b and *E. hirae* 248D) were tested in similar in vitro experiments in GM and PH medium against Ma. In GM, two strains (33B and 120B) were found to have a bactericidal effect on Ma, while one strain (248D) had a bacteriostatic effect. In addition, the *E. mundtii* 33B strain also had bactericidal activity against Ma in the PH medium. The harmful effect of acid pH on Ma was ruled out since the pH never went down below 6.8. This could imply the use of other inhibition mechanisms by strain 33B, such as competency for nutrients or bacteriocins production [16]. Although *E. hirae* strain 321A did not produce any bacteriostatic or bactericidal effect, it was able to significantly lessen Ma’s growth in the PH medium. The same effect was observed in the PH medium for *E. hirae* 120B isolated from raw sheep milk [16]. Several strains of *E. hirae* have been described as bacteriocin producers [54,55,56,57]. Globally, the evidence shows that, even though strains of *E. hirae* and *E. mundtii* with antagonistic effects against several pathogens have been described [54,55,56,57,58,59], this effect varies against Ma between isolates of these two bacterial species in sheep and goats, underlining the difficulty to isolate strains with an antimicrobial potential. Further studies would be necessary to identify the mechanism used by strain 321A to alter Ma growth.

On the other hand, we studied the interaction between Ma and CNS, given that both can be present in the mammary glands of small ruminants [28,35]. For this purpose, we used CNS strains isolated from the raw milk of healthy goats, where inflammation of the mammary gland was ruled out using the California Mastitis Test. The identification of the CNS isolates present in caprine milk as *S. petrasii* is interesting as it is only the second time that this species has been isolated in animals, both times in clinically healthy goats. This *Staphylococcus* sp. has mostly been associated with the human species; thus, inadequate milking practices have been presumed to be the reason for its presence in goat mammary secretions [37]. The importance of *Staphylococcus* as a pathogen in the mammary gland of small ruminants and its importance in public health raises a great interest in the scientific community [36,60,61,62,63]. Our results confirmed the ability of Ma to significantly grow (*p* < 0.001) in GM in a range of pH inferior to 6.67 and superior to 5.91 (Condition 1, 4 and 5; Table 7 and Table 8) regardless of the presence of *S. petrasii,* which had a steady concentration between T0 and T15 with or without the presence of Ma. This evidence shows that both bacterial species could cohabitate in the mammary gland. The ability of Ma to survive in a medium with a pH around 6 has already been described in diluted semen [11], which, as a whole, shows the resilience capacity of Ma to extracellular media with a pH between 6 and 7. However, the viability of Ma in GM was altered by the presence of *S. petrasii* 227A (Table 7), given that the concentration of Ma was significantly lower at T15 when with 227A (C5) than when without (C1) (*p* < 0.01). This shows that the replication of Ma can be negatively altered not only by LAB naturally present in milk [16] but also by other bacterial communities present in the mammary gland, such as CNS. In this sense, CNS, important pathogens of subclinical IMI in small ruminants [30], have been reported to produce bacteriocins and inhibit pathogens involved in bovine mastitis [64,65]. The different interactions observed between Ma and the two *S. petrasii* strains could be because both strains were isolated from different caprine breeds, herds (one affected by an outbreak of CA) and different geographical locations. Moreover, the results are in line with a study reporting the diversity of the milk microbiota in goats with mastitis, where both CNS and *Mycoplasma* spp. were detected in the same animal [35]. Our results increase the knowledge about the interaction between both bacterial genera that often share the same ecological niche and suggest possible influences on bacterial viability that could occur in other anatomical locations beyond the mammary gland, such as the vagina, where it has been described that both are the most abundant genera in sheep [20].

Overall, this study shows that Ma viability can be influenced by bacterial ecology. Recently, it was suggested that the presence of LAB, with antimicrobial potential, in anatomical locations where Ma can also be present could be related to the persistence of this pathogen in other anatomical sites, where LAB are not present, observed in asymptomatic carriers [16], such as the external ear canal, joints, lymph nodes or nervous system [8]. The results of the present study could support this hypothesis. Both studies suggest that, indeed, the ability of Ma to colonize and preserve itself in locations such as the mammary gland or the vagina could be influenced by the bacterial populations with which it cohabits.

*Mycoplasma agalactiae* growth was affected in the presence of wild strains of *E. hirae* (321A) and CNS (227A) isolated from meat caprine and ovine herds where antibiotic use is scarce. Indeed, the caprine herd where the dairy strain 227A was isolated in milk had never used antibiotics for the prevention or control of mastitis, and the ovine herd where the vaginal strain 321A was isolated was a herd that did not use intravaginal sponges for estrus synchronization, which are linked to a more important use of antibiotics [18,66]. Both herds were managed with a system of ecological production where the restriction on antibiotic use is mandatory. Based on this, we suggest that a lesser use of antibiotics could preserve various bacterial genera with an antagonistic effect against pathogens such as Ma. The administration of antibiotics in ovine and caprine flocks has already been suggested as a prejudicial factor to certain bacterial communities [16,18]. Moreover, these two strains were from grazing herds with scarce extensive production systems (transhumance) in Spain. Two of the three strains with an antibacterial effect against Ma previously reported [16] were from grazing meat herds that rarely used antibiotics. Moreover, in both studies, some of these strains were isolated in breeds in danger of extinction (Negra Serrana goat and Guirra ewe). The loss of these production systems and breeds could imply a loss of bacterial strains with antimicrobial potential.

## 5. Conclusions

This study describes, for the first time, different interactions of Ma with wild ovine and caprine vaginal strains of LAB and raw milk strains of CNS. A wild ovine strain of *E. hirae* (321A) and CNS (227A) were able to significantly alter the optimal growth of Ma in vitro. These antagonistic effects on Ma viability observed in this study bring further evidence to a hypothetical negative influence on the ability of Ma to colonize and replicate in a host when certain bacterial communities are present. Knowledge about the viability of Ma in the presence of different microorganisms sharing the same ecological niche could help with developing alternative treatments to reduce the use of antibiotics in small ruminants’ production.

## Figures and Tables

**Table 1:** Characteristics of the different sampled livestock.

Herd	Specie	Breed	Province	Aptitude	G
A	Caprine	Murciano-Granadina	Castellón	Dairy	No
B	Ovine	Manchega	Albacete	Meat	Yes
C	Ovine	Manchega	Albacete	Dairy	Yes
D	Ovine	Lacaune	Castellón	Dairy	No
E	Caprine	Negra-Serrana *⸸	Valencia	Meat	Yes
F	Ovine	Guirra *	Valencia	Meat	Yes
G	Ovine	Lacaune	Alicante	Dairy	No
H	Ovine	Segureña ⸸	Jaén	Meat	Yes
I	Caprine	Murciano-Granadina/Malagueña	Albacete/Murcia ^¶^	Dairy	No
J	Caprine	Malagueña	Jaén	Dairy	Yes
K	Caprine	Malagueña	Madrid	Dairy	Yes

G: grazing; *: endangered breeds; ⸸: transhumant management system; ^¶^: the farm was located on the border area between Albacete and Murcia provinces.

**Table 2:** Description of the composition of the different in vitro conditions for the vaginal LAB experiments.

Condition	Composition
C1	PH (1460 μL) + Ma (40 μL)
C2	PH (1000 μL) + LAB (500 μL)
C3	PH (960 μL) + Ma (40 μL) + LAB (500 μL)
C4	PH (1500 μL)

PH: specific medium for *Mycoplasma* spp. growth; Ma: *Mycoplasma agalactiae* strain PG2 inoculum; LAB: inoculum of each selected vaginal lactic acid bacteria strain (321A or 344A).

**Table 3:** Description of the composition of the different in vitro conditions for the *Staphylococcus* experiments.

Condition	Composition
C1	GM (1460 μL) + Ma (40 μL)
C2	GM (1000 μL) + L2 (500 μL)
C3	GM (960 μL) + Ma (40 μL) + L2 (500 μL)
C4	GM (1000 μL) + Sa (500 μL)
C5	GM (960 μL) + Ma (40 μL) + Sa (500 μL)
C6	PH (1460 μL) + Ma (40 μL)
C7	PH (1000 μL) + L2 (500 μL)
C8	PH (960 μL) + Ma (40 μL) + L2 (500 μL)
C9	PH (1000 μL) + Sa (500 μL)
C10	PH (960 μL) + Ma (40 μL) + Sa (500 μL)
C11	GM (1500 μL)
C12	PH (1500 μL)

GM: semi-skimmed UHT goat milk; Ma: *Mycoplasma agalactiae* strain PG2; L2: commercial probiotic inoculum; Sa: caprine CNS strain inoculum for each strain selected (227A and 332B); PH: specific medium for *Mycoplasma* spp. growth.

**Table 4:** Vaginal bacterial isolates per herd and selected strains.

Herd	NIS	NPS	SS	OD	C
A	27	0	-	-	-
B	34	1	-	-	-
C	40	3	-	-	-
D	17	0	-	-	-
E	14	2	-	-	-
F	19	0	-	-	-
G	20	0	-	-	-
H	16	1	321A	0.395	2.7 × 10^8^
I	10	2	344A	0.334	1.9 × 10^8^
J	14	0	-	-	-
K	3	0	-	-	-

NIS: n° of isolated strains; NPS: n° of potential strains for the experiment (good growth in PH and absence of hemolysis in Blood Agar); SS: selected strain for the experiment; OD: optical density after 20 h incubation in PH medium; C: concentration in CFU/mL after 20 h incubation in PH medium.

**Table 5:** Least square means of pH and log CFU/mL of Ma by condition and time for the experiment with vaginal strain *E. hirae* 321A.

Condition	Composition	Time (h)	321A (LOG CFU/mL) ^1^	Ma (LOG CFU/mL) ^2^	pH ^3^
C1	PH + Ma	0		6.674 ^c^	7.43 ^a^
C1	PH + Ma	15		8.665 ^a^	7.12 ^bc^
C2	PH + 321A	0	8.63 ^a^		7.16 ^b^
C2	PH + 321A	15	8.47 ^a^		7.12 ^bc^
C3	PH + Ma + 321A	0	8.43 ^a^	6.647 ^c^	7.25 ^b^
C3	PH + Ma + 321A	15	8.15 ^b^	7.892 ^b^	6.99 ^c^
C4	PH	0			7.43 ^a^
C4	PH	15			7.48 ^a^

Ma: *Mycoplasma agalactiae*; 321A: *Enterococcus hirae* strain 321A; PH: specific medium for *Mycoplasma* spp. isolation. ^a–c^: means in the same column with different superscripts between conditions and times differ significantly (*p* < 0.05). ^1^ SEM: 0.09; ^2^ SEM: 0.09; ^3^ SEM: 0.05.

**Table 6:** Least square means of pH and log CFU/mL of Ma by condition and time for the experiment with vaginal strain *E. mundtii* 344A.

Condition	Composition	Time (h)	344A (LOG CFU/mL) ^1^	Ma (LOG CFU/mL) ^2^	pH ^3^
C1	PH + Ma	0		7.067 ^b^	7.50 ^ab^
C1	PH + Ma	15		8.438 ^a^	7.20 ^cd^
C2	PH + 344A	0	8.67 ^a^		7.09 ^cd^
C2	PH + 344A	15	8.54 ^a^		6.99 ^d^
C3	PH + Ma + 344A	0	8.65 ^a^	6.778 ^c^	7.34 ^bc^
C3	PH + Ma + 344A	15	8.63 ^a^	8.365 ^a^	7.03 ^d^
C4	PH	0			7.55 ^ab^
C4	PH	15			7.67 ^a^

Ma: *Mycoplasma agalactiae*; 344A: *Enterococcus hirae* strain 344A; PH: specific medium for *Mycoplasma* spp. isolation. ^a–d^: means in the same column with different superscripts between conditions and times differ significantly (*p* < 0.05). ^1^ SEM: 0.07; ^2^ SEM: 0.08; ^3^ SEM: 0.11.

**Table 7:** Least square means of pH and log CFU/mL of Ma by condition and time for the experiment with strain 227A of *Staphylococcus petrasii*.

Condition	Composition	Time (h)	L2 (LOG CFU/mL) ^1^	227A (LOG CFU/mL) ^2^	Ma (LOG CFU/mL) ^3^	pH ^4^
C1	GM + Ma	0			7.069 ^de^	6.62 ^de^
C1	GM + Ma	15			8.430 ^b^	6.60 ^de^
C2	GM + L2	0	8.85 ^a^		-	6.50 ^e^
C2	GM + L2	15	9.04 ^a^		-	4.29 ^g^
C3	GM + Ma + L2	0	8.88 ^a^		7.116 ^d^	6.45 ^e^
C3	GM + Ma + L2	15	8.92 ^a^		0.000 ^f^	4.39 ^g^
C4	GM + 227A	0		8.28 ^abc^	-	6.82 ^cd^
C4	GM + 227A	15		8.71 ^a^	-	6.07 ^f^
C5	GM + Ma + 227A	0		8.18 ^abcd^	6.965 ^de^	6.80 ^cd^
C5	GM + Ma + 227A	15		8.56 ^ab^	8.126 ^c^	5.82 ^f^
C6	PH + Ma	0			6.812 ^e^	7.51 ^ab^
C6	PH + Ma	15			8.655 ^a^	7.33 ^b^
C7	PH + L2	0	8.94 ^a^		-	6.88 ^cd^
C7	PH + L2	15	8.95 ^a^		-	6.84 ^cd^
C8	PH + Ma + L2	0	8.92 ^a^		6.973 ^de^	6.89 ^cd^
C8	PH + Ma + L2	15	8.94 ^a^		8.128 ^c^	6.82 ^cd^
C9	PH + 227A	0		8.03 ^bcd^	-	7.41 ^ab^
C9	PH + 227A	15		7.90 ^cd^	-	6.95 ^c^
C10	PH + Ma + 227A	0		8.05 ^bcd^	7.017 ^de^	7.37 ^b^
C10	PH + Ma + 227A	15		7.57 ^d^	8.415 ^b^	6.85 ^cd^
C11	GM	0			-	6.65 ^cde^
C11	GM	15			-	6.46 ^e^
C12	PH	0			-	7.56 ^ab^
C12	PH	15			-	7.71 ^a^

GM: semi-skimmed UHT goat milk; Ma: *Mycoplasma agalactiae*; L2: commercial probiotic; 227A: coagulase-negative *Staphylococcus* strain 227A; PH: specific medium for *Mycoplasma* spp. isolation. ^a–g^: means in the same column with different superscripts between conditions and times differ significantly (*p* < 0.05). ^1^ SEM: 0.13; ^2^ SEM: 0.23; ^3^ SEM: 0.10; ^4^ SEM: 0.11.

**Table 8:** Least square means of pH and log CFU/mL of Ma by condition and time for the experiment with strain 332B of *Staphylococcus petrasii*.

Condition	Composition	Time (h)	L2 (LOG CFU/mL) ^1^	332B (LOG CFU/mL) ^2^	Ma (LOG CFU/mL) ^3^	pH ^4^
C1	GM + Ma	0			7.110 ^c^	6.66 ^efg^
C1	GM + Ma	15			8.282 ^a^	6.23 ^gh^
C2	GM + L2	0	9.02 ^b^		-	6.62 ^efg^
C2	GM + L2	15	9.36 ^a^		-	4.27 ^i^
C3	GM + Ma + L2	0	9.18 ^ab^		7.113 ^c^	6.59 ^fg^
C3	GM + Ma + L2	15	9.24 ^ab^		0.000 ^d^	4.40 ^i^
C4	GM + 332B	0		8.58 ^a^	-	6.80 ^def^
C4	GM + 332B	15		8.86 ^a^	-	5.92 ^h^
C5	GM + Ma + 332B	0		8.45 ^ab^	7.179 ^c^	6.82 ^def^
C5	GM + Ma + 332B	15		8.43 ^ab^	8.188 ^a^	6.24 ^gh^
C6	PH + Ma	0			7.060 ^c^	7.51 ^ab^
C6	PH + Ma	15			8.297 ^a^	7.36 ^abc^
C7	PH + L2	0	9.26 ^ab^		-	6.91 ^def^
C7	PH + L2	15	9.14 ^ab^		-	6.85 ^def^
C8	PH + Ma + L2	0	9.26 ^ab^		6.997 ^c^	6.94 ^cdef^
C8	PH + Ma + L2	15	9.13 ^ab^		7.495 ^b^	6.88 ^def^
C9	PH + 332B	0		8.43 ^ab^	-	7.50 ^abc^
C9	PH + 332B	15		8.28 ^ab^	-	7.06 ^cde^
C10	PH + Ma + 332B	0		8.50 ^ab^	7.023 ^c^	7.53 ^ab^
C10	PH + Ma + 332B	15		7.61 ^b^	8.214 ^a^	7.12 ^bcd^
C11	GM	0			-	6.64 ^efg^
C11	GM	15			-	6.57 ^fg^
C12	PH	0			-	7.58 ^a^
C12	PH	15			-	7.70 ^a^

GM: goat milk; Ma: *Mycoplasma agalactiae*; L2: commercial probiotic; 332B: coagulase-negative *Staphylococcus* strain 332B; PH: specific medium for *Mycoplasma* spp. isolation. ^a–i^: means in the same column with different superscripts between conditions and times differ significantly (*p* < 0.05). ^1^ SEM: 0.10; ^2^ SEM: 0.32; ^3^ SEM: 0.10; ^4^ SEM: 0.16.

## Data Availability

The data and materials are available from the corresponding author upon reasonable request. The nucleotide sequences generated in this study can be found in the GenBank repository (SUB13689910) with the following accession numbers: OR289671 (strain 227A), OR289672 (strain 332B), OR289673 (strain 321A) and OR289674 (strain 344A). The full genome sequence of strain 227A can be found in the GenBank repository (BioProject ID PRJNA1054492; SUB14096682).
